# A streamlined tandem affinity purification of His-MBP-SpyCas9, without buffer exchange, suitable for *in vitro* cleavage applications

**DOI:** 10.1016/j.mex.2025.103368

**Published:** 2025-05-12

**Authors:** Filippo Fronza, Roberto Verardo, Claudio Schneider

**Affiliations:** a DME Dipartimento di Medicina, University of Udine, Piazzale Kolbe 1, 33100 Udine, Italy; b LNCIB Laboratorio Nazionale CIB (Consorzio Interuniversitario per le Biotecnologie) BIC Incubatori, Via Flavia 23/1, 34148 Trieste, Italy; c AREA Science Park, Padriciano 99, 34149 Trieste, Italy

**Keywords:** CRISPR, Cas9, SpyCas9, SpCas9, Protein purification, His-tag, HisTRap, MBP-tag, MBPTrap, AKTA Start, FPLC, Cas9 tandem affinity purification

## Abstract

We present a streamlined protocol for the purification of recombinant doubly-tagged His-MBP-SpyCas9 protein utilizing a dual affinity chromatography approach whereby the elution volume from immobilized metal ion affinity chromatography (IMAC) is loaded directly onto maltose-binding protein (MBP) affinity chromatography. This protocol, by optimizing the buffer composition throughout the process, eliminates the need for buffer exchanges thereby reducing the risk of protein precipitation. The purification process, from bacterial harvest to final product, can be completed within a single working day. The purified Cas9 protein is suitable for *in vitro* cleavage assays as validated using sgRNA/complementary dsDNA-reporter oligonucleotides. In fact, the elution buffer from the MBP binding column is suitable for storage and the purified protein can be stored/aliquoted at -20 °C after the addition of glycerol, to be used directly *in vitro* cleavage assays without additional processing.

## Graphical abstract

**Figure:**
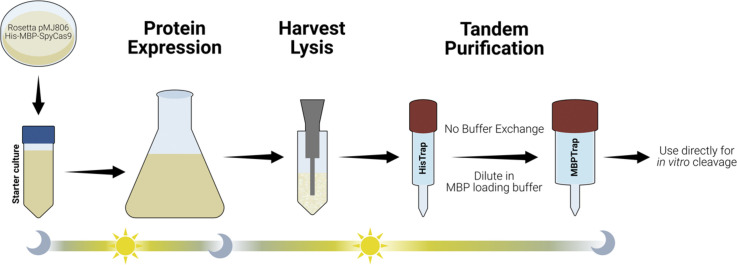

Specifications table

**Table:** 

Subject area:	Biochemistry, Genetics and Molecular Biology
More specific subject area:	Protein purification
Name of your method:	Cas9 tandem affinity purification
Name of your protocol:	A streamlined tandem affinity purification of His-MBP-SpyCas9, without buffer exchange, suitable for *in vitro* cleavage applications.
Reagents/tools:	- IPTG, isopropylthio-β-galactoside (Invitrogen, Thermo Fisher Scientific) - Kanamycin (Merk) - Chloramphenicol (Merk) - HEPES (Thermo Fisher Scientific) - Imidazole, Ultrapure (Thermo Fisher Scientific) - Glycerol, Ultrapure, HPLC Grade (Thermo Fisher Scientific)
	- NaCl, Sodium chloride (Thermo Fisher Scientific) - DTT, dithiothreitol (Thermo Fisher Scientific) - TCEP-HCL (Pierce, Thermo Fisher Scientific) - Triton X-100 (Thermo Fisher Scientific) - Halt™ Protease Inhibitor Cocktail (Thermo Fisher Scientific) - NaH2PO4, Sodium phosphate monobasic (Merk) - PMSF, Phenylmethylsulfonyl fluoride (Thermo Fisher Scientific) - TRIS, Tris(hydroxymethyl)aminomethane (Merk) - Maltose, (Merk) - LB broth, Luria (Merk): Tryptone 10 g/L, Yeast extract 5 g/L, NaCl 0.5 *g*/L - LB agar, Luria (Merk): Tryptone 10 g/L, Yeast extract 5 g/L, NaCl 0.5 g/L, Agar 15 *g*/L - Sonication buffer: 20 mM HEPES; 20 mM Imidazole, ultrapure; 300 mM NaCl; 10 % Glycerol; 0.5 mM DTT or TCEP; 1 mM TritonX-100; either Halt™ Protease Inhibitor Cocktail, a Protease Inhibitor Cocktail of your choice. - His loading buffer: 50 mM NaH2PO4; 300 mM NaCl; 20 mM Imidazole, ultrapure; 1 mM PMSF; pH8 - His high salt wash buffer: 50 mM NaH2PO4; 600 mM NaCl; 20 mM Imidazole, ultrapure; 1 mM PMSF; pH8 - His elution buffer: 50 mM NaH2PO4; 150 mM NaCl; 500 mM Imidazole, ultrapure; pH8 - MBP loading buffer: 20 mM TRIS; 200 mM NaCl; 1 mM EDTA; pH7.5 - MBP elution buffer: 20 mM TRIS; 200 mM NaCl; 10 mM Maltose; pH7.5 - Rosetta™2(DE3) Competent cells (Merk Millipore) - pMJ806 (Addgene 39,312) - ÄKTA® Start (Cytiva, Danaher) with Frac30 fractionation system - Sorvall RC-58 (Sorvall) with GS-3 and SS34 rotors - Artek Sonic Dismembrator model 301 (Artek Systems) with micrtotip probe - Filtropur BT50, vacum filtration unit, 500 mL 0.45µ m (Sarstedt) - Filtropur S, syringe filter, 0.2µ m (Sarstedt) - HisTrap HP, 1 mL (Cytiva, Danaher) - MBPTrap HP, 5 mL (Cytiva, Danaher) - Amicon® Ultra-15 100 kDa filter tubes (Merk Millipore) Validation - pUC57-sgRNA (Addgene 51,132) - T4 DNA Ligase (NEB) - Bsa-HFv2 (NEB) - STBL3 competent E.coli (Thermo Fisher Scientific) - QIAGEN plasmid plus midi kit (Qiagen) - Precision gRNA Synthesis Kit (Invitrogen, Thermo Fischer Scientific) - Reporter 1 (Supplementary) (IDT) - Reporter 2 (Supplementary) (IDT) - Reporter 3 (Supplementary) (IDT) - rCutsmart (NEB) - SYBR Green Fluorescent DNA Stain (Jena Bioscience) - PageRuler™ Plus Prestained Protein Ladder, 10 to 250 kDa (Thermo Scientific) - 100 bp DNA Ladder (NEB) - 40 % 29:1 Bis-Acrylamide (BioRad)
Experimental design:	We describe a protocol for the purification of the His-MBP-SpyCas9 protein using a two affinity chromatography setup. The protein is expressed in E.coli, lysed by sonication, purified first through a HisTrap IMAC column and then diluted and purified directly through a MBPTrap column.
Trial registration:	
Ethics:	
Value of the Protocol:	• Allows for an easier purification of Cas9 for *in vitro* applications by using a double affinity chromatographic setup with no need for buffer exchanges. In particular, the buffer of the final product do not interfere with the activity of the protein. • Because all the purification steps (from bacteria harvest to final product) can be done in a single day, there is a lower risk of protein precipitation. • The whole process is fast enough to be completed in 3 days.

## Background

Most referenced protocols for SpyCas9 purification are based on and modified from the original work of Anders, Corolin, and Jinek [1] and Lapinaite et al. [2]. where three chromatographic steps with in-between buffer exchange are used in a two-days working timeline. A cation-exchange column is always included and requires buffer exchange steps both before and after, which are procedures where the risk of protein precipitation has to be carefully taken into consideration. We describe a protocol for the purification of the His-MBP-SpyCas9 protein expressed in transformed bacteria based on a sequential tandem affinity using immobilized metal ion affinity chromatography (IMAC) nickel column for the purification of histidine-tagged protein (HisTrap HP, Cytiva) followed by a direct affinity chromatographic column for the purification of MBP-tagged protein (MBPTrap HP, Cytiva). We were able to obtain an active SpyCas9 protein at a purity suitable for *in vitro* applications without the need for buffer exchange or concentration steps.

The protocol was developed specifically to avoid the need for dialysis/buffer exchange steps and to complete both chromatographic purifications in a single day thus reducing the chance of precipitation of the protein. Moreover, because of the mild conditions of the elution from the MBP-binding column, it can be directly employed *in vitro* cleavage procedures. The SpyCas9 specific programmed nuclease activity have been validated using dsDNA oligonucleotides.

## Description of protocol

### Transformation of BLD21 (DE3) Rosetta cells

SpyCas9 protein will be expressed in *Escherichia coli* BLD21(DE3) Rosetta cells (Novagene, Merk Millipore, Burlington, Massachusetts).

1. Thaw one aliquot of commercial Rosetta cells (50µ L) on ice for 30 min.
2. Add 200 ng of pMJ806 plasmid (Addgene 39,312) to the tawed cells, and mix by pipetting.
3. Incubate on ice for 15 min.
4. Perform heat-shock: incubate at 45 °C for 45 s.
5. Incubate 3 min on ice.
6. Add 500µ L of unselective LB broth to the cells. Incubate 1 h at 37 °C.
7. Plate 100µ L of culture on selective LB agar plates supplemented with 50mg/L Kanamycin and 33mg/L Chloramphenicol.
8. Grow overnight at 37 °C to obtain colonies.

You can store the plate at 4 °C for up to 2 months for later use.

### Culture growth and protein expression

1. Prepare a starter culture by picking a colony from the plate and inoculating it in 50 mL of selective LB broth supplemented with 50mg/L Kanamycin, 33mg/L Chloramphenicol.
2. Grow bacteria overnight at 37 °C with shaking at 180 rpm.
3. Inoculate 5 mL of starter culture in 500 mL of selective LB medium (1 % v/v) in a 2 L flask. We usually prepare two 500 mL flasks at the same time.
4. Incubate at 37 °C with shaking at 180 rpm until OD_600_ 0.8–1 is reached.
5. Reduce incubation temperature to 18 °C and let the bacterial culture cool down for 30 min with shaking at 180 rpm.
6. Induce protein expression with 0.4 mM IPTG, equivalent to 2 mL of 1 M stock to 500 mL of culture.
7. Incubate overnight at 18 °C with shaking at 180 rpm.

If you want to preserve the transformed cells long-term, you can store some of the left-over starter culture after the inoculation of the main culture. Store around 1 mL of culture in 50 % glycerol at −80 °C. They can be stored for several years.

### Harvest

1. Move the culture to 500 mL polypropylene ultracentrifuge-safe bottles. Lysis will be performed in these bottles as well.
2. Harvest cells by centrifugation at 5000 rpm (∼4′200x *g*) for 10 min in a Sorvall RC-58 superspeed centrifuge (Sorvall) with a GS-3 rotor (or equivalent).
3. Discard the supernatant. Do not perturb the cell pellet.
4. Store pellet on ice.

If a supercentrifuge/ultracentrifuge is not available, harvest can be performed in a swinging bucket centrifuge at ∼2′500x g for 15–20 min. The supernatant should be clear (not cloudy) after centrifugation. To use smaller vessels, such as 50 mL centrifuge tubes, process the culture in batches and repeat the process until all culture have been pelleted.

If you want to stop, you can freeze the pellet at this stage for up to 6 months.

### Lysis by sonication

1. Resuspend cell pellets in the Sonication Buffer. The volume of the buffer should be 5 % (v/v) of the volume of the cell culture before harvest. For each 500 mL flask use 25 mL Sonication Buffer. Mix vigorously with a pipettor, vortex, or by shaking. The solution should be cloudy without clumps.
2. If you have more than one pellet, pool them together to achieve a more uniform lysis. Leave empty space in the vessel (around half) to avoid spilling over bubbles during sonication.
3. Sonicate using an immersion probe. Using an Artek Sonic Dismembrator model 301 (Artek Systems) with a microtip probe, sonicate for 1 min at 50 % power. Mix the solution gently during the process.
4. Rest lysate on ice for 1 min to maintain temperature low.
5. Repeat steps 3–4 10 to 20 times.

Since this process is highly dependent on the equipment used, it is highly recommended to adapt the procedure to ensure the best results. You may need to use shorter cycles if the solution warmings up too quickly and more cycles if the lysis is incomplete.

### Clarification of lysate

1. Transfer the solution to ultracentrifuge-safe 50 mL tubes.
2. Centrifuge at 16′000 rpm (∼30′000x g) for 30 min in a Sorvall RC-58 with a SS34 rotor (or equivalent).
3. Collect the supernatant and keep it on ice. Do not freeze it, otherwise the protein will precipitate.

If an ultracentrifuge is not available, clarification can be performed in a swinging bucket centrifuge at ∼3′000x g for 1 h.

### Purification

All chromatographic purification steps are done with an AKTA Start protein purification system (Cytiva, Danaher, Washington D.C.), or equivalent fast protein liquid chromatography (FPLC) system, at room temperature.

Before purification, filter all buffers through Filtropur BT50, 500 mL, 0.2µ m filters or equivalent, and filter the lysate through 0.2µ m syringe filters. This will avoid clogging of the chromatographic columns and FPLC instrument.

### His-tag affinity purification

The first purification step is performed with a HisTrap HP (Cytiva, Danaher, Washington D.C.) affinity chromatography column. We use the 1 mL variant running it at 1–2 mL/min with a pressure limit of 0.5 MPa

1. Prime the system initially with the His elution buffer and subsequently with the His loading buffer; this ensures that there are no bubbles in the system or lag during the elution phase.
2. Equilibrate the column in 5CV of His loading buffer.
3. Keeping 10 mL of His loading buffer at hand, start loading the lysate from the sample port. Add His lysis buffer at the end of the process to make sure that no bubbles are introduced into the system.
4. Collect fractions during and after the loading process to check them later on an SDS-PAGE.
5. Wash the column with 5CV of His loading buffer. While doing so, switch the sample port from the lysate to His high salt wash buffer.
6. Wash the column with 5CV of His high salt wash buffer from the sample port to reduce the amount of impurities in the final product.
7. Wash the column with 5CV of 6 % His elution buffer to remove additional nonspecific ligands.

During the washes you may see small peaks in the chromatogram.

**Fig. 1:**
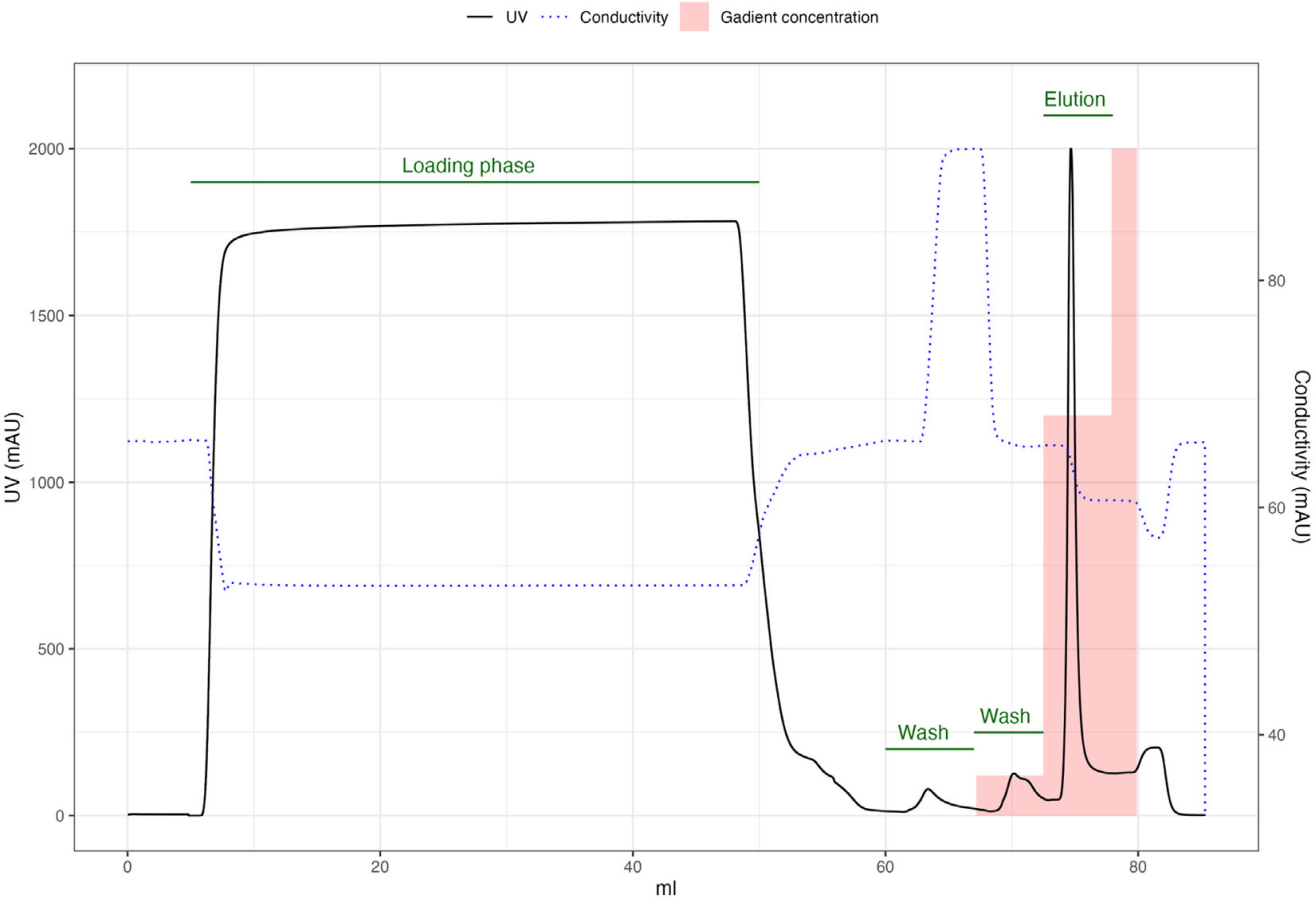
An example of the chromatogram expected during HisTrap IMAC.During the high-salt wash, imidazole wash, and post-elution wash you may observe peaks as impurities are released.

**Table 1:** Summary of His-tag purification phase.

Step	Port	Substance	Volume (CV)	Notes
Equilibration	A	His loading buffer	5	
Sample loading	Sample	Lysate	All	Add His loading buffer at the end to avoid bubbles
Wash	A	His loading buffer	5	Exchange sample port from Lysate to His high salt buffer
High salt Wash	Sample	His high salt buffer	5	
Imidazole Wash	*A* + 6 %B	His elution buffer (6 %)	5	
Elution	*A* + 60 %B	His elution buffer (60 %)	5	Fraction collected contains Cas9 protein
Post-elution wash	B	His elution buffer (100 %)	1.5	Fractions may contains protein
Wash	A	His loading buffer	5

10. Elute the Cas9 protein with 5CV 60 % His elution buffer; collect fractions with the peak elution function for the best protein concentration. Most of the protein should elute during this step.

If your system does not have peak fraction collection you can achieve a similar effect by collecting 2 mL fractions and pooling the peak fractions afterward.

11. Wash the column with 1.5CV of 100 % His elution buffer to completely remove bound proteins. You may collect fractions from this step as well.
12. Conclude the purification by washing the column in 5CV of His loading buffer.

At the end of the His-tag affinity purification process you should have 4–10 mL of eluted protein. Keep it on ice but do not freeze it to avoid precipitation.

Clean up the column following the manufacturer's instructions and store it for subsequent use.

### MBP-tag affinity chromatography

This purification step is performed in an MBPTrap HP (Cytiva, Danaher, Washington D.C.) affinity chromatography column. We use the 5 mL variant running at a flow speed of 5mL/min and a pressure limit of 0.5 MPa.

1. Prepare a volume of MBP loading buffer supplemented with 10 % (v/v) of ultrapure/HPLC grade Glycerol equal to the elution from the His purification phase.
2. Mix the elution with the buffer prepared in the previous step. Mix thoroughly.
3. Prime the system initially with the MBP elution buffer and then with the MBP loading buffer; make sure there are no bubbles in the system.
4. Equilibrate the column with 5CV of MBP loading buffer.
5. Load the sample from the sample port, and keep 10 mL of MBP loading buffer to avoid the introductions of bubbles. You may also want to reduce the flow rate to 1–2mL/min. Collect fractions for checking.
6. Wash the column with 2CV of MBP loading buffer.
7. Elute the protein with 5CV of 100 % MBP elution buffer. Collect fractions using the peak fraction function or every 2 mL and pool peak fractions afterward.
8. Wash the column with 5CV of MBP loading buffer.

**Table 2:** Summary of MBP-tag purification phase.

Step	Port	Substance	Volume (CV)	Notes
Equilibration	A	MBP loading buffer	5CV	
Sample loading	Sample	Elution from His purification	All	Add MBP loading buffer at the end to avoid bubbles
Wash	A	MBP loading buffer	2	
Elution	B	MBP elution buffer	5	Fractions collected contains Cas9 protein
Wash	A	MBP loading buffer	5

**Fig. 2:**
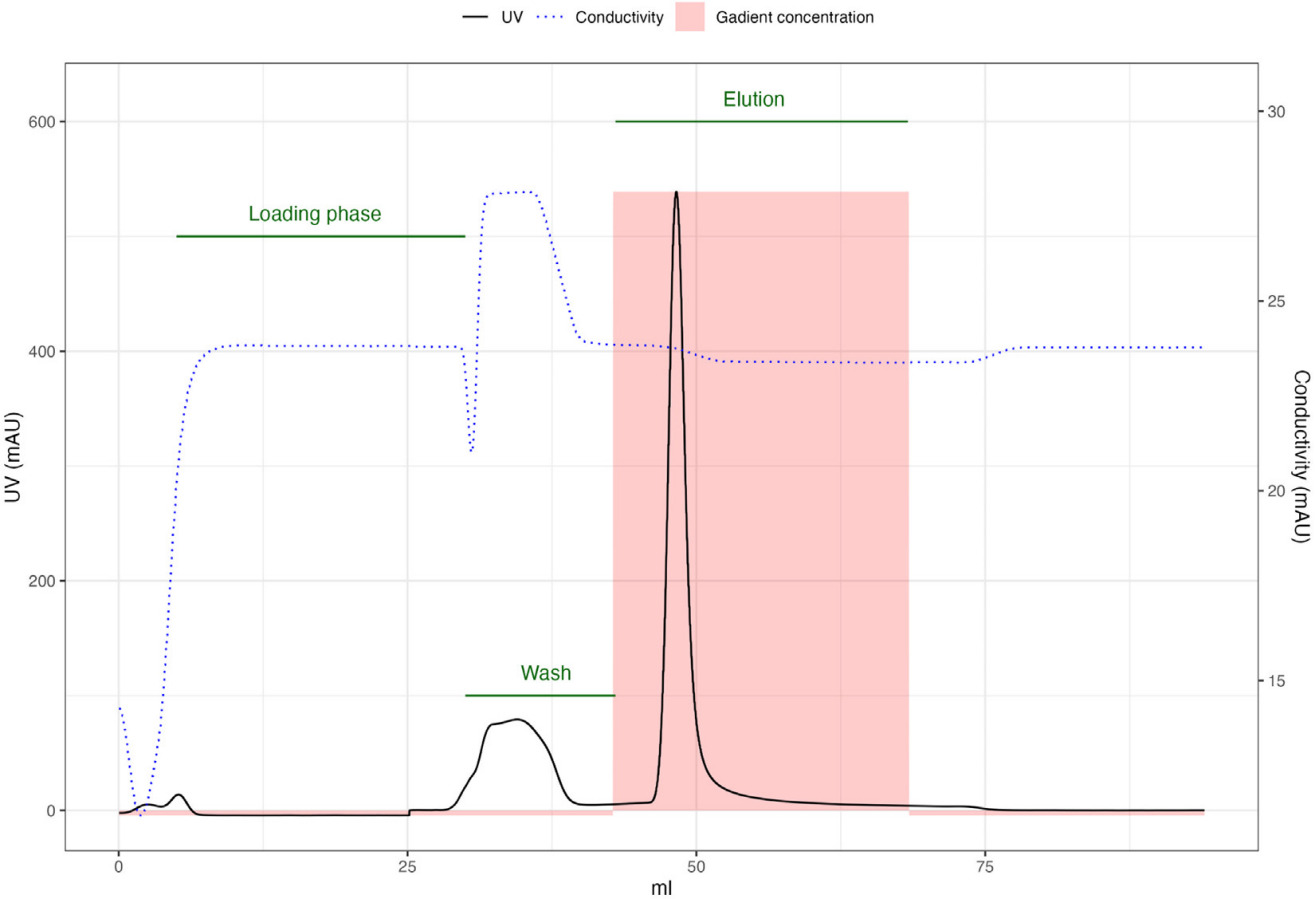
An example of the chromatogram expected during MBPTrap affinity chromatography. In this case, the UV rises after the loading phase is over because the loaded volume is lower than the volume of the column used. The unbound fraction is removed by the wash step.

**Fig. 3:**
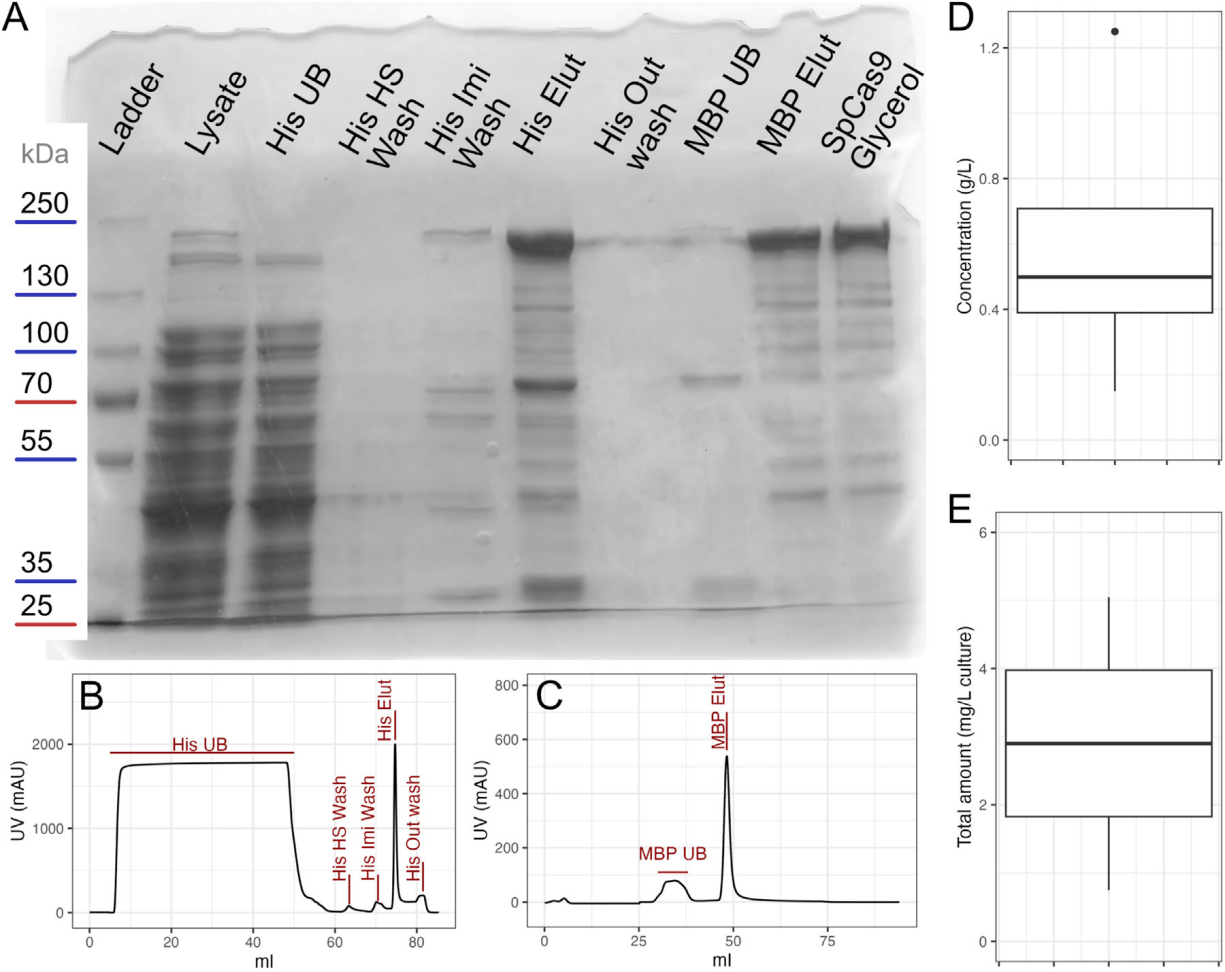
(A) SDS-PAGE of collected fractions from chromatographic steps;Ladder: PageRuler™ Plus 10 to 250 kDaLysate: initial input after sonication;His UB: unbound fraction after His loading step; His HS Wash: high salt wash of His purification;His Imi Wash: wash with 6 % His elution buffer, high in imidazole; His Elut: elution of His-tag purification, used as input to MBP purification; His Out wash: final His loading buffer wash after elution and post-elution steps; MBP UB: unbound fraction after MBP loading step, pooled with the following wash step; MBP Elut: elution of MBP purification; SpCas9 Glycerol: final product.The fractions were collected from the corresponding points in the chromatograms (B) for HisTrap purification and (C) for MBPTrap purification.The unbound fraction from MBP purification is collected during the wash phase since the volume loaded is less than the volume of the column. This is reflected in the chromatogram as the UV signal does not rise during loading but after.(D) Concentration of final fractions after addition of glycerol and (E) estimated yield per liter of bacterial culture. Panels D and E highlight results that are representative of 3 purification processes.

**Fig. 4:**
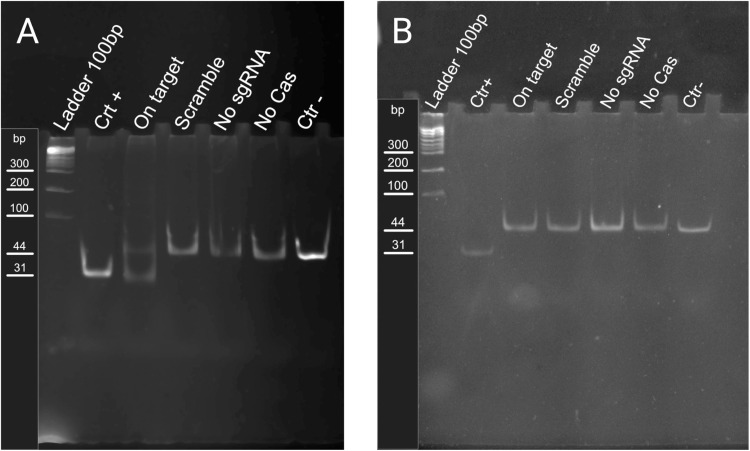
Native DNA PAGE of Reporter 1 (A) and pamless Reporter 2 (B) Ladder: 100 bp DNA Ladder Ctr +: positive control, synthetic 31 bp dsDNA that simulates cleaved long fragment; On target: reporter cleaved by Cas9 and sgRNA; Scramble: random sgRNA, negative control; No sgRNA: sgRNA is substituted with water; No Cas9: no protein or sgRNA used, substituted with MBP elution buffer + Glycerol and water; Ctr -: negative control, starting reporter dsDNA The presence of the lower bad in the on-target sample of Reporter 1 but not Reporter 2 is indicative that the protein we purified is active.

**Fig. 5:**
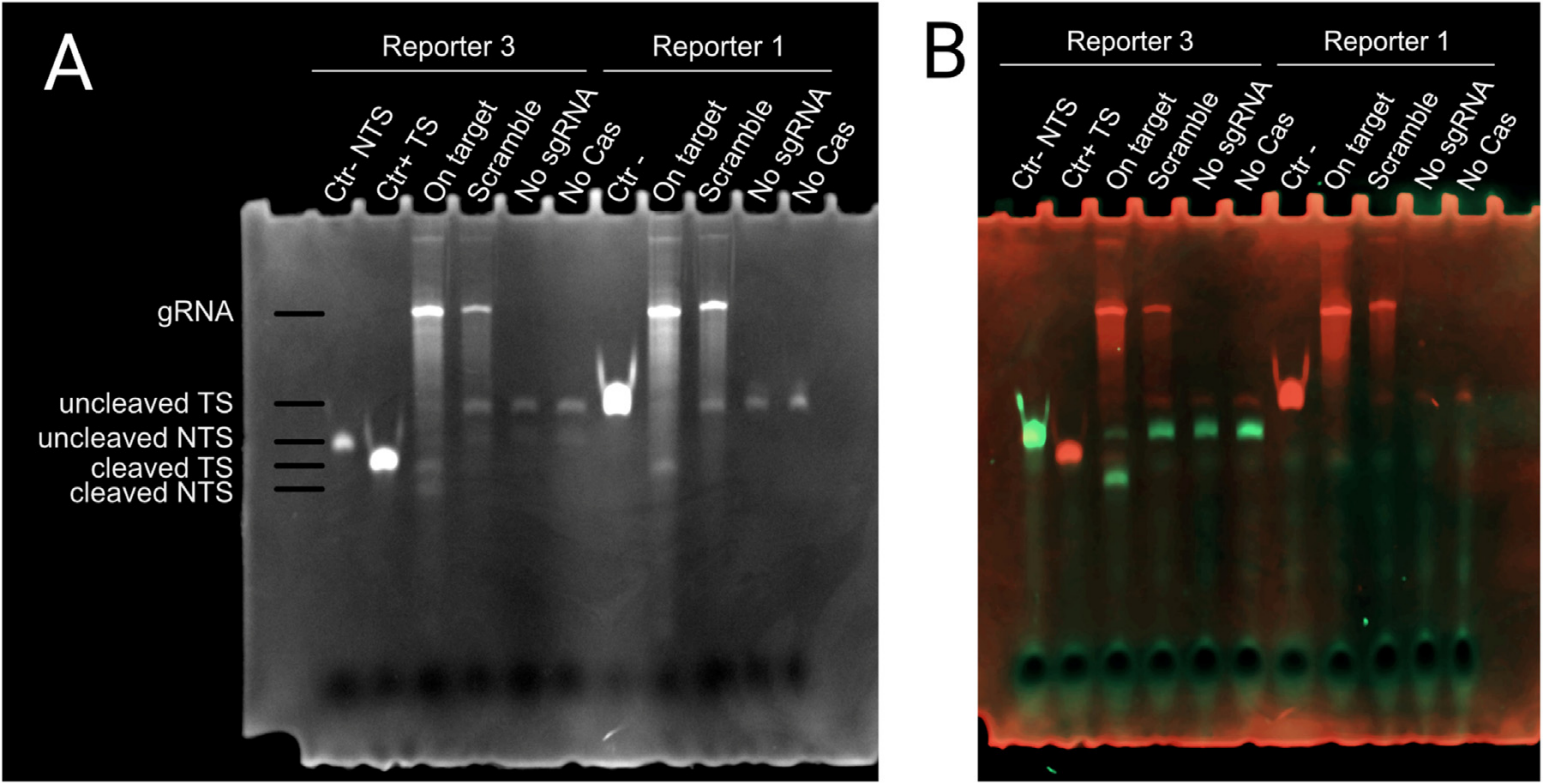
EtBr staining (A) and False color overlap of FAM and Ethidium bromide signals (B). Reporter 3 Ctr- NTS: synthetic ssDNA (IDT) labeled with FAM, negative control for NTS; Reporter 3 Ctr+ TS: synthetic ssDNA (IDT) unlabeled, positive control for cleaved TS, used for both Reporter 3 and Reporter 1; Reporter 1 Ctr-: uncleaved Reporter 1, also used as a negative control for Reporter 3 TS, which has the same size. On target: on target sgRNA, upper bands are uncleaved fraction while lower bans are cleaved fraction; Scramble: random sgRNA, negative control; No sgRNA: sgRNA is substituted with water; No Cas9: no protein or sgRNA used, substituted with MBP elution buffer + Glycerol and water; Ctr -: negative control, starting reporter dsDNA By comparing the sizes of the produced DNA fragments with the controls we can reasonably conclude the purified protein is active.

After use clean the column following manufacturer instructions for subsequent use.

### Protein storage

1. Pool the peak fractions from the MBP-tag affinity chromatography step
2. Add ultrapure/HPLC grade Glycerol to a final concentration of 40 % (v/v).

Aliquots can be stored at −20 °C for up to one year.

### Estimate of protein concentration

After MBP-purification you can estimate the protein concentration by measuring absorbance at 280 nm, using a bicinchoninic acid assay (BCA) [3], Bradford protein assay [4], or equivalent.

### (Optional) concentration

If needed, you can increase the protein concentration using Amicon® Ultra-15 100 kDa filter tubes (Merk Millipore, Burlington, Massachusetts) or equivalent.

This procedure can also be employed to remove small-size impurities or exchange the buffer if needed.

1. Above 500µ L, centrifuge in 1 min increments at ∼900x g in a swinging bucket centrifuge.
2. Mix by pipetting and repeat step 1.
3. Below 500µ L, centrifuge in 30s–1 min increments at ∼500x g.
4. Mix by pipetting and repeat step 3 until the desired concentration is achieved.

## Protocol validation

### SDS-PAGE analysis of the collected chromatographic fractions

We checked pools of the fractions from the purification steps by running 10µ L of each sample on an 8 % Acylamide SDS-PAGE gel.

Run along PageRuler™ Plus Prestained Protein Ladder, 10 to 250 kDa (Thermo Scientific, Waltham, Massachusetts).

The expected size of the His-MBP-Cas9 protein is 201.6 kD which corresponds with the topmost band in the gel. Since a Cas9 band is not apparent in the His UB fraction (see Fig. 3), most of the expressed protein is efficiently bound and eluted from the HisTrap column with a faint relative band in the His-Imidazole wash. The MBP-Trap column purification removes some of the major contaminants (MBP UB): some impurities are still present which might correspond to partial proteolysis products from the COOH-terminus. The presence of such ‘impurities’ does not affect the *in vitro* cleavage activity and neither contains non-specific DNase or RNase activities as shown in Fig. 4, Fig. 5.

### Preparation of sgRNA

Synthetic oligonucleotides for the production of the on-target sgRNA were cloned into pUC57-sgRNA plasmid (Addgene 51,132) [5] via golden gate assembly following NEB protocol [6], and transformed in competent STBL3 E.coli. Plasmid was amplified and extracted using Qiagen Plasmid Plus Midi Kit (Qiagen) and Sanger-sequenced. sgRNA was produced by *in vitro* transcription (IVT) starting from amplification by PCR of sgRNA template from plasmid, IVT and RNA purification was then performed with Invitrogen Precision gRNA Synthesis Kit (Invitrogen, Thermo Fischer Scientific). The sequence of the plasmid, of the oligonucleotides used for the golden gate assembly, and of the sgRNA are reported in Supplementary Materials.

### Activity of Cas9

We tested the activity of the purified protein *in vitro* by cleaving a synthetic dsDNA reporters (IDT) whose sequence is shown in Supplementay Materials.

The setup for the cleave reaction is the following:

• 100 nM DNA reporter
• 300 nM Cas9
• 360 nM sgRNA
• rCutSmart® buffer (NEB)
• 500 nM SYBR® Green Fluorescent DNA Stain (Jena Bioscience)
• final volume 20µ L in water.

The reaction was performed in a thermocycler, at a constant temperature of 37 °C for 5 min and then stopped by denaturation at 95 °C for 10 s.

Native PAGE was prepared following the protocol from Green et al. [7]. Gel was prepared with 15 % acrylamide, the samples were mixed with 6x Gel-Loading Buffer III described in the aforementioned protocol, and run at 80 V in a 10 cm long gel until the Bromophenol Blue band reached 3/4 of the gel length.

Samples were run along with 100 bp DNA Ladder (NEB, Ipswich, Massachusetts), to which were added 500 nM final concentration of SYBR Green I before loading.

We performed the same reaction using a normal reporter, Reporter 1, and a reporter without the appropriate PAM sequence, Reporter 2, to make sure that bands appearing in the on-target sample were due to specific cleavage.

We also performed the cleavage assay using a reporter labeled with FAM on one end of the non-target strand (NTS) and an elongated target strand (TS), Reporter 3. After the reaction, the samples were run without the addition of dyes on a denaturing urea polyacrylamide gel (7 M Urea, 15 % Acrylamide) following the protocol from Summer et al. [8].

The first picture taken after electrophoresis is without staining and shows only the FAM-labeled strands (Supplementary Figure 1). The gel was then fixed (50 % Methanol, 10 % Acetic acid) for 90 min, washed three times in water, and stained (Ethidium Bromide 0.5 mg/ml, TAE buffer 1x) for 30 min and then washed three times in water (Fig. 5). EtBr was used for the staining because it can stain both DNA and RNA with a high sensitivity.

## Limitations

This protocol requires an FPLC instrument and works best with an AKTA Start or equivalent with a fraction collection system because the ability to collect fractions specifically from the peaks of the elution increases the concentration of the final product.

The purity of the final product is not going to be the highest that can be obtained. This protocol is a ``good enough'' approach for applications in which very high protein purity is not required.

The protein purified by this protocol has not been tested for use in cell cultures.

## Supplementary material *and/or* additional information [OPTIONAL]

Supplementary file PUR01_supplementary.docx contains additional figures to the validation process.

Supplementary file PUR01_supp_sequences.xlsx contains the sequence of the reporter oligonucleotides used, of the sgRNA, and oligonucleotides used for the golden gate assembly method. puc57-sgrna.fasta and puc57-sgrna-on-target.fasta contains the sequence of the plasmids used to produce sgRNAs.

## CRediT authorship contribution statement

**Filippo Fronza:** Conceptualization, Investigation, Methodology, Validation, Visualization, Writing – original draft. **Roberto Verardo:** Conceptualization, Methodology, Resources, Supervision, Writing – review & editing. **Claudio Schneider:** Conceptualization, Funding acquisition, Methodology, Resources, Supervision, Writing – review & editing.

## Declaration of competing interests

The authors declare that they have no known competing financial interests or personal relationships that could have appeared to influence the work reported in this paper.

## Data Availability

Data will be made available on request.

## Appendix Supplementary materials

Image, application 1

Image, application 2

Image, application 3

Image, application 4
