# Supplementary material for: A streamlined tandem affinity purification of His-MBP-SpyCas9, without buffer exchange, suitable for in vitro cleavage applications
Source: MethodsX. 2025 May 12;14:103368. doi: 10.1016/j.mex.2025.103368 (PMC12143660; doi:10.1016/j.mex.2025.103368)
Supplement: Supplementary file 4 [file mmc4.docx]

Supplementary:


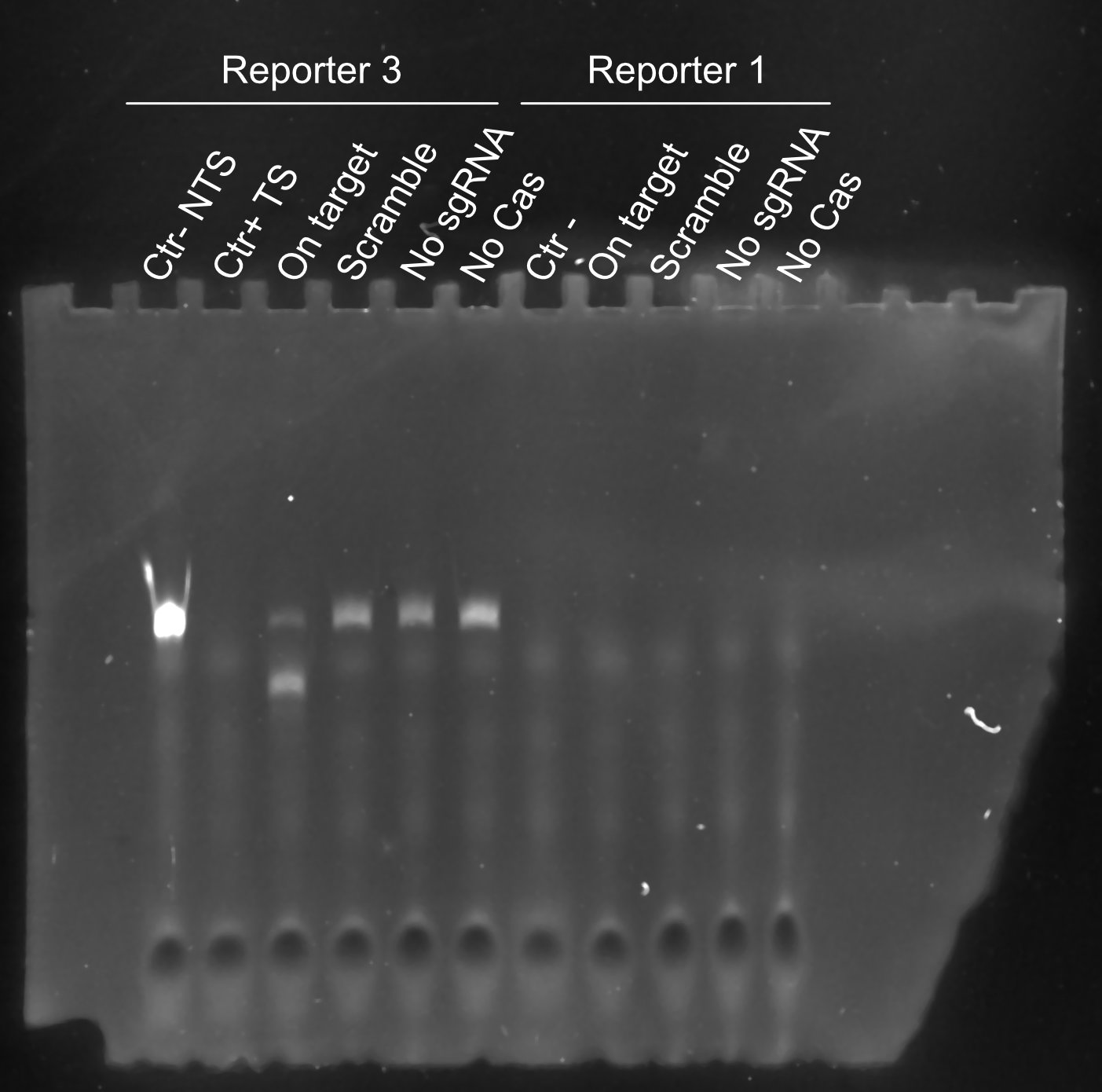
Figure 1: FAM signal of Reporter 3 before EtBr staining.Since Reporter 1 is not fluorescent on its own, as expected no signal is observed.
